# Effects of Nutritional Supplementation on Tumor Growth: A Systematic Review and Meta-Analysis of Studies Using Animal Models of Mammary Cancer

**DOI:** 10.3390/biology15020150

**Published:** 2026-01-14

**Authors:** Bruna Ribeiro-Silva, José Antônio Orellana Turri, Ricardo dos Santos Simões, José Cipolla-Neto, Edmund Chada Baracat, José Maria Soares-Jr

**Affiliations:** 1 Disciplina de Ginecologia, Departamento de Obstetrícia e Ginecologia, Hospital das Clínicas da Faculdade de Medicina da Universidade de São Paulo, São Paulo 17012-901, SP, Brazil; brunaribeirobrf@outlook.com (B.R.-S.); ricardo.simoes@hc.fm.usp.br (R.d.S.S.); 2 Laboratório de Ginecologia Estrutural e Molecular (LIM 58) do Departamento de Obstetrícia e Ginecologia, Faculdade de Medicina da Universidade de São Paulo, São Paulo 17012-901, SP, Brazil; antonioturri@usp.br; 3 Laboratório de Neurobiologia do Departamento de Fisiologia, Instituto de Ciências Biomédicas, Universidade de São Paulo, Av. Lineu Prestes, 1524, Bldg 1, São Paulo 05508-000, SP, Brazil; cipolla@icb.usp.br

**Keywords:** animal model with walker 256 cells, nutritional supplement, mammary tumor growth, systematic review with meta-analysis

## Abstract

Breast cancer remains the most prevalent malignancy among women worldwide, and experimental tumor models are essential for evaluating new therapeutic approaches before clinical application. The Walker-256 carcinoma model exhibits biological behavior comparable to human carcinoma and allows short-term assessment of tumor progression. This study aimed to conduct a systematic review and meta-analysis of the effects of dietary supplementation on Walker-256 tumor growth. We followed the PRISMA guidelines and searched the PubMed, Embase, and Cochrane databases without time restrictions. The meta-analysis included clustering based on the type of supplementation received, comparing treatment groups with control groups. Twenty-one studies were included, analyzing 18 different supplements, and 12 studies were considered for quantitative synthesis. The overall effect indicated a significant reduction in tumor growth with supplementation (SMD = 2.83, 95% CI = 1.99–3.66, *p* < 0.001), despite high heterogeneity (I^2^ = 93.3%). Oils had the greatest impact, particularly fish oil (SMD = 6.99, 95% CI = 3.15–10.83, *p* < 0.001) and shark liver oil (SMD = 4.43, 95% CI = 2.19–6.67, *p* < 0.01).

## Simple Summary

Breast cancer is the most common type of cancer affecting women worldwide. To develop new treatments, researchers often use experimental tumor models before clinical studies. The Walker-256 tumor model is commonly used because it shares similarities with human carcinoma and grows quickly, allowing efficient testing. In this systematic review and meta-analysis, we examined 21 studies and performed a detailed analysis of 12 of them to investigate whether dietary supplements could reduce tumor growth. We found that many supplements, particularly fish oil and shark liver oil, significantly reduced tumor growth. Other supplements, including amino acids and those rich in linoleic acid, also showed promising effects. We conclude that nutritional supplementation has the potential to reduce tumor growth in Walker-256 models.

## 1. Introduction

Breast cancer is the most common malignancy among women worldwide [1] and is a leading cause of cancer-related mortality [2]. According to Azamjah et al. [3], between 1990 and 2015, breast cancer mortality rates increased in Latin America and the Caribbean. Due to increasing mortality rates from breast cancer, adjuvant strategies, like dietary supplementation, have been investigated for their potential to decrease tumor growth [4]. Several experimental studies have employed the Walker-256 model to evaluate the effects of dietary supplementation on tumor growth. This experimental model for tumor induction in rodents exhibits biological behavior similar to that of human carcinomas and enables short-duration studies (12–16 days) [5,6,7]. Initially described by Dr. George Walker, the tumor arises spontaneously in the left mammary gland of a pregnant rat. The original tumor consisted of an inferior portion that was hemorrhagic and necrotic, with free blood-tinged fluid, and a superior portion with a granular, reddish-gray appearance containing small foci of necrosis [8] model to evaluate the effects of dietary supplementation on tumor growth.

Through successive inoculations and technical improvements, Walker-256 cells replicate rapidly in host rodents and are maintained in the laboratory via weekly intraperitoneal inoculations. Seven days after inoculation, animals are anesthetized and euthanized. Cells are harvested from the abdominal cavity, centrifuged, suspended in phosphate-buffered saline or Hank’s balanced salt solution, and tested for viability using the Trypan Blue exclusion assay in a Neubauer chamber before being stored for reinoculation [9,10,11,12,13,14,15,16].

For studies using this model, a solid tumor is obtained, and abnormal tissue masses are classified as either carcinoma or sarcoma according to their histopathological characteristics. To prevent bacterial contamination, the cell suspension obtained from the peritoneal cavity is combined with antibiotics, such as benzylpenicillin, and inoculated into mice either subcutaneously or intramuscularly, usually in the flank region, but also potentially in the femur, tibia, paw, thigh, or armpit [5,7,13,15,17]. Tumor growth is rapid, with masses reaching an average diameter of 20–30 mm in approximately eight days [11,18], making the model suitable for preclinical oncology investigations. However, there is no consensus regarding the optimal number of cells to be inoculated [5].

Although several studies have used the Walker-256 carcinoma to evaluate the effects of dietary supplementation on tumor growth, no comprehensive consolidation of these findings is currently available. Therefore, this systematic review and meta-analysis aimed to gather and critically analyze the current evidence regarding the impact of various dietary supplements on tumor growth in the Walker-256 carcinoma model, thereby strengthening preclinical knowledge in this field.

## 2. Materials and Methods

This systematic review was conducted in accordance with the PRISMA Statement guidelines [19] and was prospectively registered in the International Prospective Register of Systematic Reviews (PROSPERO; registration number CRD42025639872). The PRISMA 2020 checklist is attached to this research as Supplementary Material.

We included studies that used the Walker-256 carcinoma model to compare solid tumor growth in rats receiving nutritional supplementation with tumor growth in non-supplemented controls with no limitations concerning the supplement categories or the site of tumor implantation. Eligible publications were written in English, Portuguese, Spanish, French, or Italian, with no restriction on publication date.

We excluded systematic reviews; studies with methodological inadequacies; studies with missing information on dosage, sample size, or experimental duration; studies that combined supplementation with other drugs; studies using parenteral nutrition, intragastric administration, or gastric tube feeding; long-term supplementation across one or more generations; studies involving pregnant rats; and studies evaluating tumor growth in offspring of supplemented animals.

The selection of studies was carried out in the PubMed, Embase, and Cochrane databases; searches were also conducted in the gray literature, but no additional eligible studies were identified. The materials were accessed according to the eligibility criteria. The search strategy used Medical Subject Headings terms, Embase Thesaurus, and free terms and was completed in October 2025. The search strategy is described in Supplementary Material (Table S1: PICO strategy; Table S2: PubMed search strategy; Table S3: Embase search strategy; Table S4: Cochrane search strategy).

Study selection was blinded and performed in two stages by two reviewers (B.R.-S. and J.M.S.-J.): (1) screening of titles and abstracts and (2) full-text review to identify experiments using murine models of Walker-256 carcinoma that compared solid tumor growth in supplemented and non-supplemented groups. Discrepancies were identified and resolved through discussions with a third author (R.d.S.S.).

The following information was extracted from each eligible study: first author, year of publication, number of cells inoculated, inoculation site, rat strain, experimental duration, sample size, type of dietary supplementation, country of origin, and outcomes related to tumor growth. Numerical data were initially collected from tables, text, and figures; when values were not explicitly reported, they were estimated from graphs using digital ruler software (ImageJ, National Institutes of Health, version 1.53). Two reviewers (B.R.-S. and J.M.S.-J.) independently extracted the data and discrepancies were resolved by discussion with a third reviewer (R.d.S.S.). If data remained unclear or unavailable, corresponding authors were contacted via email (up to two attempts). For outcomes measured at multiple time points, the time point showing the greatest efficacy was included in the analysis. Tumor growth was summarized as the percentage difference in final tumor weight between control and experimental groups and standardized on a 0–5 scale for descriptive and comparative purposes in Table 1 only; this scale was not used in effect-size calculations and does not influence the meta-analytic results. All quantitative meta-analyses were performed using the extracted continuous data (mean ± standard deviation) of final tumor weights.

Meta-analysis was performed to evaluate the effect of dietary supplementation on tumor growth. The primary outcome was tumor growth reduction, assessed by comparing the mean and standard deviation (SD) of final tumor weights between experimental and control groups. Analyses were conducted by grouping studies according to the type of supplement, with further subcategories defined as amino acid, coconut oil, fish, shark liver, high linoleic acid, and high oleic acid.

For descriptive analysis, means, SDs, mean differences, and 95% confidence intervals (CIs) were calculated. The percentage weight of each study was determined for the overall standardized analysis. All statistical analyses were performed using STATA 16-SE, with a significance level of 5%. Heterogeneity was assessed using Higgins’ I^2^ statistic. Forest plots were generated to synthesize results of comparable studies. Effect sizes represented the efficacy of each supplementation in reducing tumor growth compared with controls, with pooled effect sizes indicated by red diamonds. Dashed black lines denoted the line of null effect, and the width of the red diamonds corresponded to the combined CI for each supplement group. Estimates crossing the null line were interpreted as showing no efficacy or lacking statistical significance.

Risk of bias was evaluated using SYRCLE’s risk of bias tool for animal studies [41], adapted from the Cochrane Risk of Bias tool (Figure S1: Risk of bias). Domains included selection, performance, detection, and reporting bias, with emphasis on study design. Two reviewers (B.R.-S. and J.M.S.-J.) independently extracted data, and any discrepancies were resolved through discussion or consultation with a third reviewer.

## 3. Results

**Figure 1:**
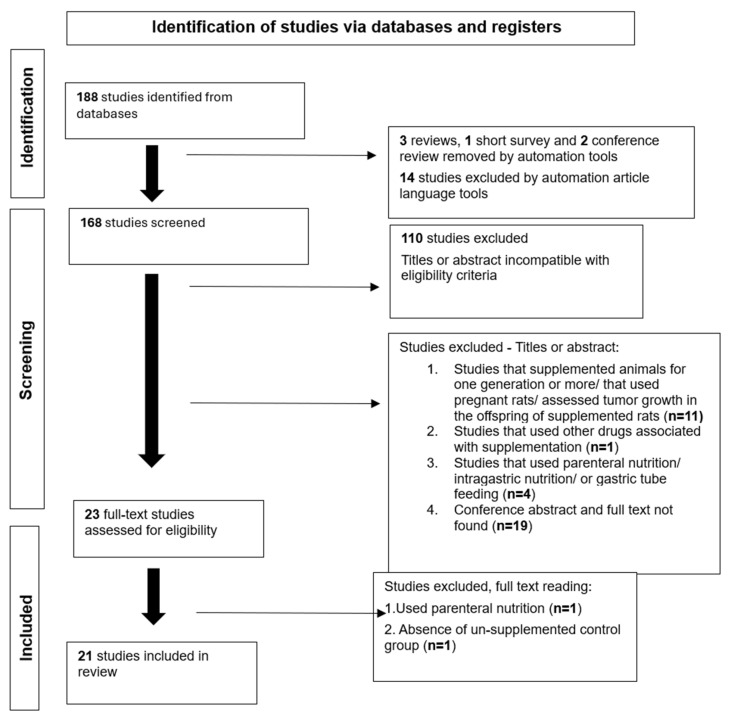
Flowchart of the search for and selection of articles used in the present systematic review.

A total of 188 articles were identified in the database; 165 were excluded after title/abstract screening and 2 after full-text review, resulting in 21 eligible studies [20,21,22,23,24,25,26,27,28,29,30,31,32,33,34,35,36,37,38,39,40]. The article selection process is depicted in Figure 1.

Most studies utilized Wistar murine, with an average experimental duration of 39 days (SD: 25.922 days), and the mean number of animals per study was 58 (SD: 41.622). For tumor induction, the average number of cells used was 18,553 × 10^3^ (SD: 25,888 × 10^3^), except for studies [36,37,38,40], which reported using 100 ± 3 mg of tumor tissue, equivalent to approximately 50 to 100 × 10^3^ tumor cells. Most studies administered injections to the flank of the animals.

The analyzed studies were conducted in Brazil, the United Kingdom, and the United States, with the majority performed in Brazil. The supplements investigated included honey, aloe vera, creatine, leucine (L), chia, fish oil (FO), Sacha inchi oil (Inca oil), shark liver oil (SO), glutamine (GL), coconut oil, sunflower oil, gamma-linolenic acid (GLA), soybean oil, corn oil, almond oil, macadamia oil, cod liver oil, and medium-chain triglycerides (MT), with fish oil being the most extensively investigated. All studies that used leucine as a supplement incorporated 3% leucine into the diet, whereas those that used glutamine incorporated 4% glutamine into the diet. Honey and aloe vera were provided at a dose of 670 μL/kg/day. The oils—coconut, fish, Oro Inca, and shark liver oil—were provided at 1 g/kg/day, with the exception of the study by Nathalia Pizato et al. (2005) [35], which incorporated 198 g/kg of fish oil as well as sunflower oil into the diet. In the study by Alison Colquhoun et al. (1998) [38], supplementation was provided at 0.4% of body weight per day.

Tumor growth parameters were assessed as a secondary outcome in studies by Cruz et al. (2017) [23], Salomão et al. (2010) [24], Colquhoun (2002) [36], and Colquhoun et al. (1998) [38]. An increase in tumor growth was observed in two studies [25,35], which used chia and coconut oil, respectively, as supplements. A significant reduction in tumor growth percentage was observed in 13 studies, 7 of which employed fish oil [28,29,30,31,33,34,35]. In addition to fish oil, Schiessel et al. (2015) [28] used Inca oil, and Pizato et al. (2005) [35] used sunflower oil; both showed reductions in tumor growth when used independently. Shark liver oil demonstrated a growth-reducing effect in three studies [29,31,32]. Other supplements such as soybean oil [37,38], corn oil [39], almond oil, cod liver oil [37], and gamma-linolenic acid [36,37] also demonstrated tumor growth reduction.

In eight studies, no significant changes were observed with supplementation. Leucine showed a minimal significant effect on tumor reduction in three studies [22,23,25] and a non-significant reduction in two others [24,26]. Salomão et al. (2010) [24] combined supplementation with regular physical activity (30 min daily for 10 days) and analyzed glutamine both alone and in combination with leucine. Creatine [21], macadamia oil [38], and medium-chain triglycerides [40] showed reduction effects below 10%, which were considered neutral. Fabiola Iagher et al. (2011) [32] reported that coconut oil supplementation did not produce significant changes in tumor growth, and when combined with shark liver oil, although it showed a reduction compared to the unsupplemented group, the effect was smaller than with shark liver oil alone. The combination of fish oil and shark liver oil (Fabiola Iagher et al., 2013) [29] resulted in a significant reduction in tumor growth, while the combination of fish oil and sunflower oil [35] had a neutral effect.

### 3.1. Meta-Analysis Results

A meta-analysis was conducted on studies evaluating the effect of dietary supplements on tumor growth reduction. The studies were grouped by supplement type and classified into two major categories: oils (*n* = 8 studies) and amino acids (*n* = 4 studies). These were further divided into the following subgroups: amino acids, coconut oil, fish oil, shark liver oil, oils rich in linoleic acid (soybean, corn, sacha inchi, and sunflower oils), and oils rich in oleic acid (macadamia, almond, and cod liver oils). The amino acid group included leucine and glutamine.

The study by Carnier et al. (2018) [27] was excluded from the analysis because chia was considered an isolated type of supplementation, as were honey and aloe vera [20]. Seven additional studies were excluded because they did not report the standard deviation of either the initial or final tumor weight, or the mean tumor weight at any time point, to avoid potential data bias. These studies were Laís Viana et al. (2019) [22], Cella et al. (2020) [21], Sérgio Belo et al. (2010) [31], Mund et al. (2007) [34], Colquhoun (2002) [36], J. Black et al. (1994) [39], and Kenneth Fearon et al. (1985) [40].

### 3.2. Overall Effect of Supplementation on Tumor Growth Reduction

**Figure 2:**
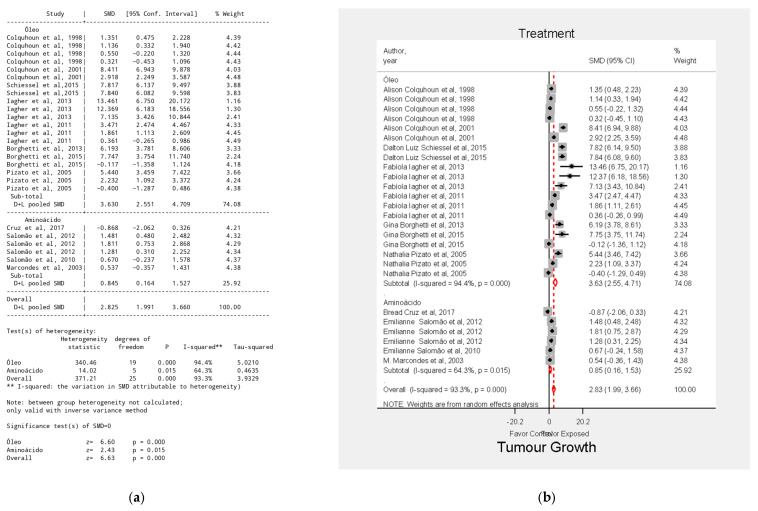
Overall effect of supplementation on tumor growth (**a**) Table of the meta-analysis general results; (**b**) Forest plot of the meta-analysis general results. Data were derived from the included studies [23,24,25,26,28,29,30,32,33,35,37,38].

The results indicated that supplementation significantly affected tumor growth. The overall effect size was SMD = 2.83, 95% CI = 1.99–3.66, *p* < 0.001 (Figure 2). The substantial variability in supplementation types, tumor induction doses, number of animals, experiment duration, and combined supplement use likely contributed to the high heterogeneity observed.

### 3.3. Effect of Amino Acids on Tumor Growth Reduction

**Figure 3:**
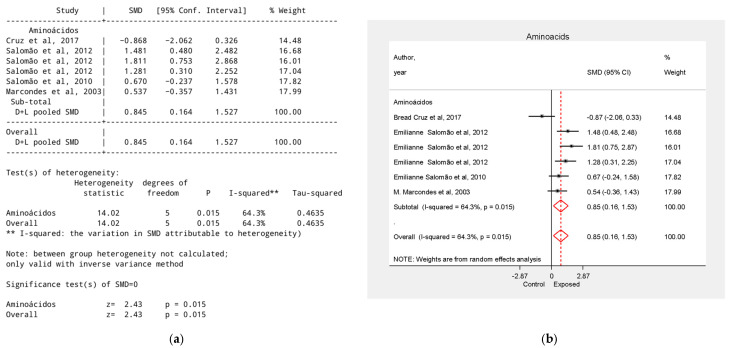
Effect of Amino Acids on Tumor Growth Reduction. (**a**) Table of the meta-analysis results in the amino acids group; (**b**) Forest plot of the meta-analysis in the amino acids group. Data were derived from the studies [23,24,25,26].

Leucine used alone showed heterogeneous results: three studies [24,25,26] reported moderate to high tumor reduction (SMDs = 1.21, 0.67, and 0.53), while one study [23] found an increased tumor effect (SMD = −0.87, 95% CI = −2.06 to 0.33). However, analyses [23,24,25] did not show statistically significant results. One study [26] analyzed the effect of glutamine, reporting a very high effect when used alone (SMD = 1.81, 95% CI = 0.75 to 2.87) and when combined with leucine (SMD = 1.28). The overall effect size for this group was SMD = 0.85, 95% CI = 0.16 to 1.53, *p* < 0.05 (Figure 3).

### 3.4. Effect of Oleic Acid on Tumor Growth Reduction

**Figure 4:**
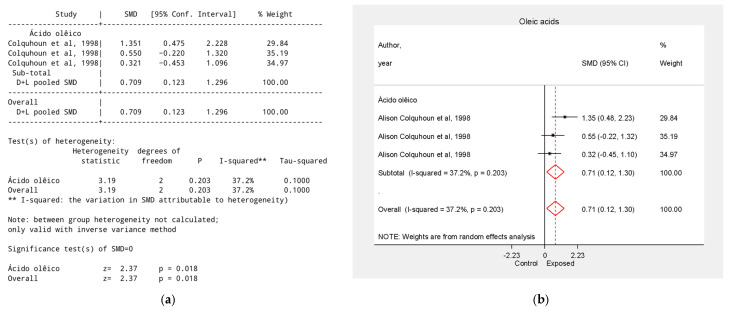
Effect of Oleic Acid on Tumor Growth Reduction. (**a**) Table of the meta-analysis results in the oleic acid group; (**b**) Forest plot of the meta-analysis in the oleic acid group. Data were derived from the study [38].

A single study [38] analyzed three supplements’ rich in oleic acid, with an overall effect size of SMD = 0.71, 95% CI = 0.12 to 1.30, *p* < 0.05. Macadamia oil supplementation (SMD = 0.32, 95% CI = −0.45 to 1.10) and almond oil (SMD = 0.55, 95% CI = −0.22 to 1.32) showed no statistically significant effect (Figure 4).

### 3.5. Effect of Linoleic Acid–Rich Supplements on Tumor Growth Reduction

**Figure 5:**
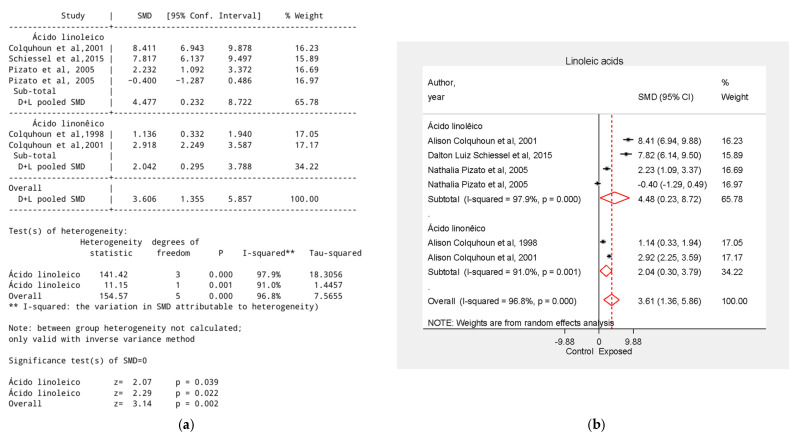
Effect of Linoleic Acid–Rich Supplements on Tumor Growth Reduction. (**a**) Table of the meta-analysis results in the linoleic acid group; (**b**) Forest plot of the meta-analysis in the linoleic acid group. Data were derived from the studies [28,35,37,38].

Essential fatty acids rich in linoleic acid—such as Inca oil, sunflower oil and their derivative gamma-linolenic acid [28,35,37,38]—showed a strong overall effect (SMD = 4.48), as well as soybean oil [37,38] (SMD = 2.04). Sunflower oil [35] showed tumor reduction (SMD = 2.23) when used independently, the analysis of the same supplement when combined with fish oil did not show statistical significance (SMD = −0.40, 95% CI = −1.29 to 0.49). Inca oil [28] showed an SMD = 7.82, 95% CI = 6.14 to 9.88, and gamma-linolenic acid [37] SMD = 8.41, 95% CI = 6.94 to 9.88 (Figure 5).

### 3.6. Effect of Coconut Oil on Tumor Growth Reduction

**Figure 6:**
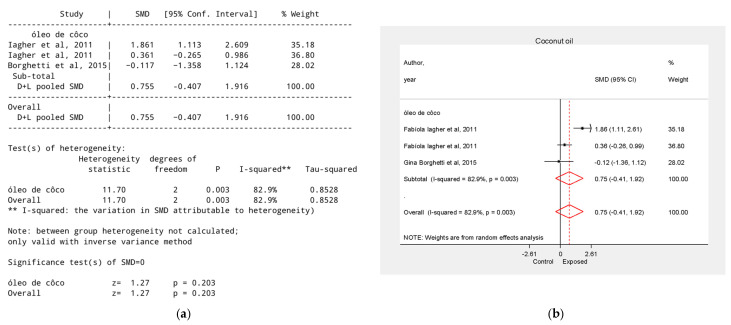
Effect of Coconut Oil on Tumor Growth Reduction. (**a**) Table of the meta-analysis results in the coconut oil group; (**b**) Forest plot of the meta-analysis in the coconut oil group. Data were derived from the studies [32,33].

One study [32] analyzed coconut oil in combination with shark liver oil, reporting SMD = 1.86, 95% CI = 1.11 to 2.61. The meta-analysis (Figure 6) did not reveal a statistically significant difference between groups (SMD = 0.73, 95% CI = −0.41 to 1.92, *p* > 0.05).

### 3.7. Effect of Fish Oil on Tumor Growth Reduction

**Figure 7:**
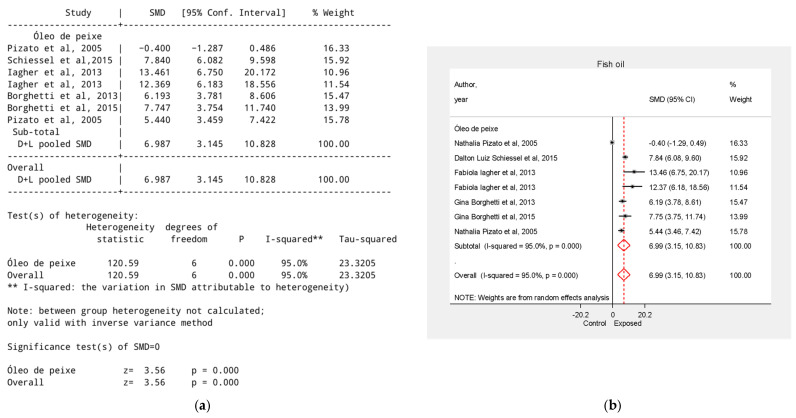
Effect of Fish Oil on Tumor Growth Reduction. (**a**) Table of the meta-analysis results in the fish oil group; (**b**) Forest plot of the meta-analysis in the fish oil group. Data were derived from the studies [28,29,30,32,33,35].

The overall effect of fish oil was SMD = 6.99, 95% CI = 3.15 to 10.83, *p* < 0.001. Two studies evaluated fish oil combined with other supplements—sunflower oil [35] and shark liver oil [29]—reporting SMD = −0.40, 95% CI = −1.29 to 0.49 and SMD = 12.37, 95% CI = 6.18 to 18.56, respectively (Figure 7).

### 3.8. Effect of Shark Liver Oil on Tumor Growth Reduction

**Figure 8:**
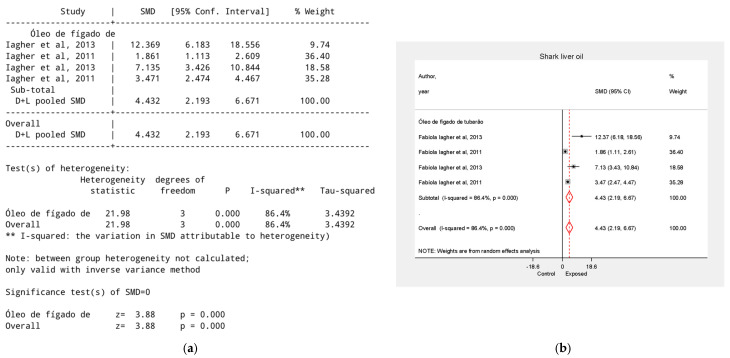
Effect of Shark Liver Oil on Tumor Growth Reduction. (**a**) Table of the meta-analysis results in the shark liver oil group; (**b**) Forest plot of the meta-analysis in the shark liver oil group. Data were derived from the studies [29,32].

Shark liver oil was analyzed in two studies by Fabiola Iagher et al. [29,32]. The overall group effect was SMD = 4.43, 95% CI = 2.19 to 6.67, *p* < 0.01. Shark liver oil was administered in combination with coconut oil [32] (SMD = 1.86, 95% CI = 1.11–2.61) and fish oil [29] (SMD = 12.37, 95% CI = 6.18–18.56). When administered alone, shark liver oil yielded SMD values of 3.47 and 7.14, (Figure 8). 

## 4. Discussion

The antitumor effects of various oils and supplements were evaluated in this study based on preclinical evidence. Fish oil, which is rich in n-3 polyunsaturated fatty acids (EPA and DHA), demonstrated the most consistent effect in reducing tumor growth. Seven studies [28,29,30,31,33,34,35] reported a reduction of more than 42% in tumor volume, with statistically significant differences, suggesting its potential as an adjuvant in breast cancer treatment. Study [35] indicated a negative interaction between fish oil and sunflower oil, as their combination resulted in a maintenance effect, whereas both supplements individually exhibited tumor growth reduction effects.

Shark liver oil, rich in alkylglycerols, also showed positive effects, though less pronounced than fish oil, and the combination of both oils did not display an additive effect. Studies indicate that the effects of these oils on tumor growth are associated with reduced tumor cell proliferation, increased apoptosis, and enhanced lipid peroxidation in tumor cells [29,32]. The SMD values that deviated most from the overall mean in the groups analyzing fish oil, shark liver oil, coconut oil, and sunflower oil corresponded to studies that tested supplements in combination with others, which contributed to the high heterogeneity observed in these groups.

Among vegetable oils rich in linoleic acid, soybean and sunflower oils possess similar properties, with linoleic acid comprising more than 50% of their composition [42]. In contrast, Plukenetia volubilis (Inca peanut) oil [28] contains approximately 35% linoleic acid and 44% alpha-linolenic acid (ALA) [43]. ALA is not present in high concentrations in the other supplements in this group, which may explain its more pronounced protective effect. The linoleic acid derivative gamma-linolenic acid [37] also demonstrated a significant protective effect, possibly related to the accumulation of poorly metabolized acyl-CoAs in tumor cell cytoplasm. Combined with changes in mitochondrial composition, membrane potential, and ultrastructure, this accumulation may promote apoptosis [37].

Oils rich in oleic acid, such as macadamia, almond, and cod liver oils, exhibited modest effects; the proportion of oleic acid in each supplement was 60.7%, 70.2%, and 23.9%, respectively [38]. Although cod liver oil had a lower oleic acid percentage than the others, it showed better results, possibly due to its mixed composition that includes n-3 fatty acids. Notably, a single study [38] evaluated all three types of oils mentioned, and in that analysis, macadamia oil did not show a statistically significant difference. Among the amino acids evaluated, leucine was the primary supplement studied. The proposed mechanism for its antitumor effect involves modulation of tumor cell metabolism, favoring oxidative phosphorylation over aerobic glycolysis and thus reducing glucose consumption by tumor cells [22].

Overall, the findings indicate that dietary supplements—particularly fish oil—have the potential to significantly reduce tumor growth in the Walker-256 carcinoma model. This systematic review and meta-analysis is the first to comprehensively evaluate the influence of supplementation on tumor growth in the Walker-256 model, yielding promising results across various types of supplements; however, additional research is required to determine their potential translation into clinical practice. Notably, high SMD values (>7) were observed, which may be associated with the characteristics of controlled experimental designs and animal models, as well as the relatively large mean differences between intervention and control groups in relation to the pooled standard deviation, which may contribute to inflated SMD estimates in preclinical studies with small sample sizes.

Although nutritional interventions may hold potential relevance in oncology, clinical translation requires caution. The findings of this meta-analysis should be interpreted in light of its limitations. Most notably, we observed extremely high heterogeneity among studies (I^2^ > 95%), which was not fully resolved by subgroup analysis. This heterogeneity likely stems from methodological differences across preclinical studies, including variations in supplement formulations, experimental timelines, and tumor induction protocols. While indicative of a general trend, the pooled effect size should be considered with caution. Given that the present review has an exploratory nature and provides an initial mapping of nutritional supplements tested in the Walker-256 model, such heterogeneity was expected, and future research would benefit from standardized experimental models and reporting to minimize such variability. Additionally, the current analysis did not include sensitivity analyses or meta-regression to formally explore sources of heterogeneity, which is an important avenue for further investigation.

The overall certainty of the evidence was also limited by methodological shortcomings identified in the risk-of-bias assessment. Many studies lacked essential details on allocation procedures, housing control, and blinding, resulting in an intermediate risk of bias across several SYRCLE domains. Although random allocation was frequently reported, methods were seldom described, and information on age or weight matching was often incomplete. In addition, some studies did not explain exclusions or discrepancies in the number of animals analyzed, indicating a potential risk of bias. Another point to consider is the predominance of Brazilian studies, which, while reflecting strong research engagement with the Walker-256 model, may introduce location bias. These issues may have reduced confidence in the pooled estimates. Future well-controlled experimental and clinical studies are needed to confirm these effects and clarify their relevance in human cancer biology.

## 5. Conclusions

This systematic review and meta-analysis indicates that dietary supplements—fish oil, shark liver oil, sacha inchi oil, gamma-linolenic acid, soybean oil, sunflower oil, cod liver oil, leucine and glutamine—may have the potential to significantly reduce tumor growth in the Walker-256 carcinoma model. However, the findings should be interpreted with caution due to the substantial heterogeneity observed among the included studies.

## Figures and Tables

**Table 1:** Features extracted from the articles in this study.

Supplement	Author, Year	No. of Cells (×10^6^)	Tumor Site	Experiment Duration (Days)	No. of Animals	Strain	Country	Tumor Growth
Honey and Aloe Vera	Rebeka Tomasin et al., 2024 [20]	5	NS	21	20	MW	BR	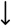 (2)
Creatine	Paola Cella et al., 2020 [21]	70	RF	21	16	MW	BR	-
Leucine	Laís Viana et al., 2019 [22]	2.5	RF	20	16	FW	BR	-
Leucine	Bread Cruz et al., 2017 [23]	1	NS	21	72	FW	BR	-
Leucine	Emilianne Salomão et al., 2010 [24]	0.25	RF	81	93	MW	BR	-
Leucine	Maria Marcondes et al., 2003 [25]	0.25	RF	12	36	MW	BR	-
GL + L Glutamine Leucine	Emilianne Salomão et al., 2012 [26]	0.25	RF	14	80	MW	BR	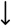 (1) 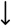 (1) 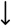 (1)
Chia	Marcela Carnier et al., 2018 [27]	20	RF	70	28	MW	BR	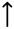 (4)
FO Oro Inca	Dalton Schiessel et al., 2015 [28]	30	RF	42	144	MW	BR	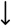 (5) 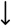 (5)
FO + SO FO SO	Fabiola Iagher et al., 2013 [29]	30	RF	70	30	MW	BR	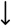 (5) 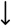 (5) 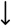 (3)
FO	Gina Borghetti et al., 2013 [30]	30	RF	44	17	MW	BR	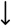 (5)
FO SO SO + FO	Sérgio Belo et al., 2010 [31]	30	RF	85	40	MW	BR	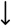 (5) 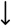 (4) 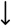 (4)
SO Coconut SO + CCO	Fabíola Iagher et al., 2011 [32]	30	RF	63	120	MW	BR	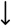 (3) - 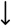 (1)
Coconut FO	Gina Borghetti et al., 2015 [33]	100	RF	45	15	MW	BR	- 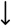 (4)
FO Coconut	Rogéria Mund et al., 2007 [34]	20	RF	63	70	MW	BR	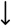 (4) 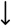 (3)
FO Sunflower oil SFO + FO	Nathalia Pizato et al., 2005 [35]	20	RF	70	70	MW	BR	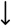 (5) 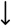 (2) -
GLA	Alison Colquhoun, 2002 [36]	0.1	Flanks	12	72	MW	BR	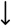 (4)
GLA Soybean oil	Alison Colquhoun et al., 2001 [37]	0.1	Flanks	12	40	MW	BR	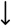 (4) 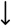 (2)
Soybean oil Almond oil Macadamia oil Cod liver oil	Alison Colquhoun et al., 1998 [38]	0.05	Flanks	12	67	MW	BR	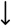 (2) 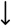 (1) - 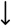 (1)
Corn oil	Jane Black et al., 1994 [39]	0.01	right thigh	23	150	MSD	EUA	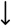 (3)
MT	Kenneth Fearon et al., 1985 [40]	0.1	RF	21	24	FW	UK	-

Note: 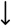 = Tumor reduction or 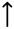 = increase in tumor growth, it was classified as follows in parenthesis: 10–21% = (1), 22–31% = (2), 32–41% = (3), 42–51% = (4), 52–61% = (5). Values below 10% were interpreted as maintenance of tumor growth, represented by: -. NS = no specified, RF = Right flank, MW = Male Wistar, FW = Female Wistar, MSD = Male Sprague-Dawley, BR = Brazil, FO = Fish oil, SO = Shark liver oil, GL + L = Glutamine e Leucine, SO + CCO = Shark liver oil e Coconut oil, SFO + FO = Sunflower oil e Fish oil, GLA = Gamma-linolenic acid, MT = medium-chain triglycerides.

## Data Availability

No new data were created or analyzed in this study. All data used in this systematic review and meta-analysis are derived from previously published studies. Data sharing is not applicable.

## Supplementary Materials

The following supporting information can be downloaded at: https://www.mdpi.com/article/10.3390/biology15020150/s1: PRISMA 2020 checklist, Table S1: PICO strategy; Table S2: PubMed search strategy; Table S3: Embase search strategy; Table S4: Cochrane search strategy; Figure S1: Risk of bias. No additional data, analytic code, or other materials were produced beyond those reported in the main text and supplementary files of this review.

## Abbreviations

The following abbreviations are used in this manuscript:

FW	Female Wistar
NS	No specified
MSD	Male Sprague-Dawley
BR	Brazil
EUA	United States
UK	United Kingdom
FO	Fish oil
SO	Shark liver oil
GL	Glutamine
L	Leucine
CCO	Coconut oil
SFO	Sunflower oil
GLA	Gamma-linolenic acid
MT	Medium-chain triglycerides
MW	Male Wistar
SD	Standard deviation
SMD	Standardized mean difference
CI	Confidence interval
PRISMA	Preferred Reporting Items for Systematic Reviews and Meta-Analyses
PROSPERO	International Prospective Register of Systematic Reviews
I^2^	Higgins’ heterogeneity statistic
SE	Standard error
HBSS	Hank’s Balanced Salt Solution
PBS	Phosphate-buffered saline
