# Supplementary material for: Effects of Nutritional Supplementation on Tumor Growth: A Systematic Review and Meta-Analysis of Studies Using Animal Models of Mammary Cancer
Source: Biology (Basel). 2026 Jan 14;15(2):150. doi: 10.3390/biology15020150 (PMC12837550; doi:10.3390/biology15020150)
Supplement: Supplementary file 1 [file biology-15-00150-s001.zip › spm.pdf]

Supplementary Materials

Search strategy

This study addresses the following research question: *Does nutritional supplementation influence tumor growth reduction in rodent models of Walker-256 carcinoma compared to non-supplemented controls?*

**PICO Element**	**Description**	**Component**
P	Population	Rodents bearing Walker-256 tumor
I	Intervention	Nutritional supplementation
C	Comparison	Control group without supplementation
O	Outcome	Reduction in tumor growth

Table S1. PICO strategy.

MeSH Terms
(Carcinoma 256, Walker OR Walker Carcinoma 256 OR Carcinosarcoma 256, Walker OR Walker Carcinosarcoma 256) AND (Dietary Supplements OR Dietary Supplementations OR Supplementations, Dietary OR Food Supplementations OR Food Supplements OR Food Supplement OR Supplement, Food OR Supplements, Food OR Nutraceuticals OR Nutraceutical OR Nutriceuticals OR Nutriceutical OR Neutraceuticals OR Neutraceutical OR Herbal Supplements OR Herbal Supplement OR Supplement, Herbal OR Supplements, Herbal OR Fatty Acids, Omega-3 OR Fatty Acid, n3 OR n3 PUFA OR PUFA, n3 OR n3 Polyunsaturated Fatty Acid OR n3 Oils OR Omega 3 Fatty Acids OR Fish Oils OR Oil, Fish OR Fish Liver Oils OR Liver Oils, Fish OR Diet OR Leucine OR Leucine, L-Isomer OR Leucine, L Isomer OR L-Isomer Leucine)

Table S2. PubMed search strategy.

#1 ('walker carcinoma'/exp OR 'walker carcinoma' OR 'carcinoma 256 walker' OR 'walker carcinoma 256') AND [embase]/lim n= 1,674<br><br>#2 ('dietary supplement'/exp OR 'dietary supplement' OR 'dietary supplementations' OR 'nutraceutical'/exp OR 'nutraceutical' OR 'herbal supplements' OR 'herbal supplement'/exp OR 'herbal supplement' OR 'n3 pufa' OR 'n3 oils' OR 'omega 3 fatty acids' OR 'fish oils' OR 'fish oil'/exp OR 'fish liver oils'/exp OR 'fish liver oils' OR 'leucine'/exp OR 'leucine') AND [embase]/lim n = 221.858<br><br>Study #1 AND #2 : n = 72

Table S3. Embase search strategy.

#1(Carcinoma 256, Walker OR Walker Carcinoma 256 OR Carcinosarcoma 256, Walker OR Walker Carcinosarcoma 256) n= 1<br><br>#2(Dietary Supplements OR Dietary Supplementations OR Supplementations, Dietary OR Food Supplementations OR Food Supplements OR Food Supplement OR Supplement, Food OR Supplements, Food OR Nutraceuticals OR Nutraceutical OR Nutriceuticals OR Nutriceutical OR Neutraceuticals OR Neutraceutical OR Herbal Supplements OR Herbal Supplement OR Supplement, Herbal OR Supplements, Herbal OR Fatty Acids, Omega-3 OR Fatty Acid, n3 OR n3 PUFA OR PUFA, n3 OR n3 Polyunsaturated Fatty Acid OR n3 Oils OR Omega 3 Fatty Acids OR Fish Oils OR Oil, Fish OR Fish Liver Oils OR Liver Oils, Fish OR Diet OR Leucine OR Leucine, L-Isomer OR Leucine, L Isomer OR L-Isomer Leucine) n = 113646<br><br>Study #1 AND #2 : n = 0

Table S4. Cochrane search strategy.

Risk of bias

We assessed the risk of bias in the included studies using the Systematic Review Center for Laboratory Animal Experimentation (SYRCLE) from the Cochrane Risk of Bias tool. The assessed domains included selection, performance, detection, and reporting biases, with a focus on study design. There were some concerns regarding potential bias in outcome measurement. All studies reported random allocation, but the exact method was not described; therefore, they were classified as having an intermediate risk for Domain 1.

In Domain 2, all studies reported that the animals within each group were of the same breed and sex. Only the studies by Tomasin et al. (2024), Cella et al. (2020), Colquhoun (2002), Colquhoun et al. (2001), Colquhoun et al. (1998), and Black et al. (1994) did not report the animals ages, whereas the others described animals of the same age. Cella et al. (2020), Colquhoun (2002), Colquhoun et al. (2001), Colquhoun et al. (1998), and Black et al. (1994) reported the similarity in rodent weights.

In the selected studies, it was not stated whether allocation was concealed from the investigator who assigned the animals, nor were there details regarding randomization of housing. Most studies, however, reported collective housing under controlled temperature, humidity, and light–dark cycles. No study provided detailed information on blinding during supplementation administration or on random selection of animals for outcome analysis. Considering these factors, all studies presented an intermediate risk of bias in Domains 3, 4, 5, and 6.

All studies showed a low risk of bias in Domains 9 and 10, as they reported the outcomes planned in their methodology, adhered to ethical and regulatory standards, and did not appear to have funding or conflicts of interest influencing the study design. Only the studies by Tomasin et al. (2024) and Cella et al. (2020) explicitly reported blinding of the outcome assessor; therefore, the remaining studies were considered to have an intermediate risk for Domain 7.

In the studies by Tomasin et al. (2024), Cella et al. (2020), Viana et al. (2019), Carnier et al. (2018), Borghetti et al. (2015), Schiessel et al. (2015), Iagher et al. (2013), Borghetti et al. (2013), Iagher et al. (2011), Salomão et al. (2010), Mund et al. (2007), Pizato et al. (2005), Marcondes et al. (2003), Colquhoun (2002), Colquhoun et al. (2001), Colquhoun et al. (1998), and Fearon et al. (1985), the number of animals varied by outcome, but the criteria for exclusion or loss were not explained, indicating a high risk of bias in Domain 8.

Overall, the studies were considered to have an intermediate risk of bias, as the lack of clear and detailed methodological information limited the strength of the evidence provided.

Risk of bias											
Study	D1	D2	D3	D4	D5	D6	D7	D8	D9	D10	Overall
Tomasin et al., 2024 (22)	⊖	✔	⊖	⊖	⊖	⊖	✔	⊖	✔	✔	⊖
Cella et al 2020 (23)	⊖	✔	⊖	⊖	⊖	⊖	✔	✖	✔	✔	⊖
Viana et al, 2019 (24)	⊖	✔	⊖	⊖	⊖	⊖	⊖	✖	✔	✔	⊖
Carnier et al, 2018 (25)	⊖	✔	⊖	⊖	⊖	⊖	⊖	⊖	✔	✔	⊖
Cruz et al, 2017 (26)	⊖	✔	⊖	⊖	⊖	⊖	⊖	✔	✔	✔	⊖
Borghetti et al, 2015 (27)	⊖	✔	⊖	⊖	⊖	⊖	⊖	✖	✔	✔	⊖
Schiessel et al, 2015 (28)	⊖	✔	⊖	⊖	⊖	⊖	⊖	✖	✔	✔	⊖
Iagher et al, 2013 (29)	⊖	✔	⊖	⊖	⊖	⊖	⊖	✖	✔	✔	⊖
Borghetti et al, 2013 (30)	⊖	✔	⊖	⊖	⊖	⊖	⊖	✖	✔	✔	⊖
Salomão et al, 2012 (31)	⊖	✔	⊖	⊖	⊖	⊖	⊖	✔	✔	✔	⊖
Iagher et al,2011 (32)	⊖	✔	⊖	⊖	⊖	⊖	⊖	✖	✔	✔	⊖
Belo et al,2010 (33)	⊖	✔	⊖	⊖	⊖	⊖	⊖	✔	✔	✔	⊖
Salomão et al, 2010 (34)	⊖	✔	⊖	⊖	⊖	⊖	⊖	✖	✔	✔	⊖
Mund et al, 2007 (35)	⊖	✔	⊖	⊖	⊖	⊖	⊖	✖	✔	✔	⊖
Pizato et al, 2005 (36)	⊖	✔	⊖	⊖	⊖	⊖	⊖	⊖	✔	✔	⊖
Marcondes et al, 2003 (37)	⊖	✔	⊖	⊖	⊖	⊖	⊖	✖	✔	✔	⊖
Colquhoun, 2002 (38)	⊖	✔	⊖	⊖	⊖	⊖	⊖	✖	✔	✔	⊖
Colquhoun et al, 2001 (39)	⊖	✔	⊖	⊖	⊖	⊖	⊖	✖	✔	✔	⊖
Colquhoun et al, 1998 (40)	⊖	✔	⊖	⊖	⊖	⊖	⊖	✖	✔	✔	⊖
Black et al,1994 (41)	⊖	✔	⊖	⊖	⊖	⊖	⊖	✔	✔	✔	⊖
Fearon et al,1985 (42)	⊖	✔	⊖	⊖	⊖	⊖	⊖	✖	✔	✔	⊖

D1) Was the allocation sequence adequately generated and applied?
D2) Were the groups similar at baseline or were they adjusted for confounders in the analysis?
D3) Was the allocation to the different groups adequately concealed during?
D4) Were the animals randomly housed during the experiment?
D5) Were the caregivers and/or investigators blinded from knowledge which intervention each animal received during the experiment?
D6) Were animals selected at random for outcome assessment?
D7) Was the outcome assessor blinded?
D8) Were incomplete outcome data adequately addressed?
D9) Are reports of the study free of selective outcome reporting?
D10) Was the study apparently free of other problems that could result in high risk of bias?

Judgement:

✔ : Low risk of bias

⊖ : Unclear/ intermediarium risk of bias

✖ : High risk of bias

Figure S1. Risk of bias.
